# Roles of Amino Acid Properties in Regulating the Gel Characteristics of Low-Salt Pacific White Shrimp (*Litopenaeus vannamei*) Surimi

**DOI:** 10.3390/foods15020400

**Published:** 2026-01-22

**Authors:** Yiting Gu, Wanying Sun, Jiao Jia, Jianan Yan, Bin Lai, Haitao Wu, Ce Wang

**Affiliations:** 1 Key Laboratory of Aquatic Product Processing and Quality Control, SKL of Marine Food Processing & Safety Control, National Engineering Research Center of Seafood, Collaborative Innovation Center of Seafood Deep Processing, School of Food Science and Technology, Ministry of Agriculture and Rural Affairs, Dalian Polytechnic University, Dalian 116034, China; 15142246566@163.com (Y.G.); tbz426304@163.com (J.J.); yjn3vv@163.com (J.Y.); laibin@dlpu.edu.cn (B.L.); wht205@163.com (H.W.); 2 Liaoning Key Laboratory of Lignocellulose Chemistry and BioMaterials, Liaoning Collaborative Innovation Center for Lignocellulosic Biorefinery, College of Light Industry and Chemical Engineering, Dalian Polytechnic University, Dalian 116034, China; 13190219563@163.com

**Keywords:** amino acids, low salt, shrimp surimi gel, gel properties, in vitro digestion, myofibrillar proteins

## Abstract

To improve the gel quality of low-salt shrimp surimi gel (SSG) from Pacific white shrimp (*Litopenaeus vannamei*), L-arginine (L-Arg), L-lysine (L-Lys), and L-proline (L-Pro) were used as partial substitutes for NaCl. The effect of the three amino acids on gel properties, protein conformation, microstructure, and in vitro digestion of low-salt SSG were systematically analyzed. Macro-/microstructural analyses revealed that L-Arg, L-Lys, and L-Pro promoted denser three-dimensional networks in low-salt SSG with smaller pore sizes. Compared with the low-salt control (LC) group, the addition of L-Arg, L-Lys, and L-Pro significantly increased the gel strength of low-salt SSG. Cooking loss was significantly decreased from 10.80% (LC group) to 1.89–4.31%. Protein solubility and turbidity results demonstrated that all amino acids markedly enhanced protein solubilization and inhibited protein aggregation. L-Arg and L-Lys mainly promoted hydrogen and disulfide bonds, but reduced hydrophobic interactions and ionic bonds. L-Arg impaired digestibility only in the gastric phase, whereas L-Lys suppressed digestibility across both gastric and intestinal phases. Through molecular docking technology, ASN-238 and LYS-187 of myosin (the dominant gel-forming protein) are the key shared binding residues with three amino acids. These findings suggest that amino acids provide a feasible approach to specifically modulate the gel characteristics of low-salt surimi products.

## 1. Introduction

Pacific white shrimp (*Litopenaeus vannamei*), renowned for its high protein content and polyunsaturated fatty acids, along with other bioactive compounds, is highly favored by global consumers [1]. According to statistics, Pacific white shrimp accounts for over 70% of global shrimp production, which magnifies its crucial role in the aquaculture industry [2]. Shrimp surimi products are fabricated from fresh or frozen shrimp with salt and other ingredients, chopped, shaped, and reheated to form a gelatinized food [3]. The global surimi industry exemplifies a powerful model for successful resource utilization. Having reached a market value of USD 3.78 billion in 2022, the market is anticipated to expand at a compound annual rate of 6.1% through 2030 [4]. Gel formation in restructured shrimp surimi is highly dependent on the ionic strength contributed by salt [5]. Typically, shrimp surimi products contain 2–3% salt to facilitate the dissolution of salt-soluble myofibrillar proteins [6]. These myofibrillar proteins form a cohesive matrix and establish a robust three-dimensional network structure through heat-induced aggregation and cross-linking, consequently triggering gelation [7]. However, excessive sodium intake (>5 g/d in adults) has been firmly linked to multiple health issues, elevating the risks of hypertension, heart disease, stroke, and many other diseases [8,9]. The Global Burden of Disease study points to a substantial rise in global cardiovascular burden over the coming decades. Its estimates suggest that from 2025 to 2050, case numbers may grow by 90%, potentially causing up to 35.6 million deaths by 2050 [10]. Moreover, according to the World Health Organization, modifying the formulations of processed foods represents a viable approach to lowering sodium consumption, which aligns with the global target of achieving a 30% reduction in dietary salt/sodium intake [11]. Therefore, the food industry faces the challenge of reducing sodium content in shrimp surimi products without compromising their quality. However, reducing salt content hinders the gelation process by impairing myofibrillar protein solubility, resulting in weaker gels with diminished water-holding capacity and instable structure [12]. Consequently, the imperative challenge lies in reducing sodium content while retaining gel functionality and the quality of shrimp surimi-based products.

In recent years, amino acids have been employed to reduce sodium in meat and surimi products, and this has aroused considerable interest [13]. Amino acids constitute the basic building blocks of proteins, acting as an innocuous, abundantly accessible, and economically sustainable food additives [14]. Research has reported that positively charged alkaline amino acids, for instance L-arginine (L-Arg) and L-lysine (L-Lys), could interact with negatively charged residues of myosin molecules through electrostatic interactions. This interaction disrupts intramolecular and intermolecular ionic bonds, modifies the myosin structure, and thus enhances the solubility of myosin in low-salt solutions [15]. L-proline (L-Pro) could increase tilapia myosin solubility in low-salt environments, and promote the formation of oligomeric aggregates from myosin particles [16]. Currently, investigations into the effects of amino acids on surimi gelation have largely focused on a limited selection of alkaline amino acids. However, there has been no research to systematically compare the effects of various amino acid classes (alkaline amino acids and cyclic amino acid) on the gelation behavior of reduced-sodium surimi-based systems, specifically shrimp surimi. Such a comparison would broaden the selection of green additives as salt substitutes. Moreover, the degree of gelation in shrimp surimi products affects their digestibility, such as residence time in the stomach and nutrient release rate, which in turn influences intestinal absorption efficiency and overall nutritional value [17]. However, the impact of amino-acid-induced gel property alterations on the gastrointestinal digestibility of low-salt shrimp surimi gel (SSG) remains unreported. Furthermore, the application of Raman spectroscopy to elucidate microenvironmental changes in low-salt SSG remains unexplored. Therefore, this study will explicate the distinct mechanism of three specific amino acids (L-Arg, L-Lys, and L-Pro) in low-salt SSG by explaining their unique side chain groups: the guanidinium group, the ε-amino group, and the cyclic ring, respectively. Cluster analysis will also be applied to investigate the variability between samples and clarify their relationships. This research aims to bridge the molecular-to-macroscopic gap by constructing a mechanistic pathway that connects protein conformation, network structure, and ultimate gel performance.

To address these knowledge gaps, three amino acids were incorporated into low-salt SSG to evaluate their effects on key gel properties, including gel strength, texture, cooking loss, water mobility, microstructure, and in vitro simulated digestibility. Changes in intermolecular forces and protein conformation in low-salt SSG were assessed using chemical force analysis, Fourier transform infrared spectroscopy, and Raman spectroscopy. The pH, protein solubility, and turbidity were used to reveal the effects of various amino acids on shrimp myofibrillar proteins. Finally, the interaction mechanism between shrimp myosin and amino acids was elucidated by molecular docking analysis, and the underlying relationships within the gel characteristic indicators of low-salt SSG were elucidated using principal component analysis. This research will establish a robust theoretical framework for designing reduced-sodium shrimp surimi-based food catering to individuals possessing specific nutritional needs, thereby broadening the application prospects of amino acids in food technology.

## 2. Materials and Methods

### 2.1. Materials and Chemicals

Pacific white shrimp (*Litopenaeus vannamei*) was sourced from Dalian Qianhe Market. Sodium chloride was purchased from Dalian Salt Industry Co., Ltd. (Dalian, China). L-Arg, L-Lys, and L-Pro were of analytical grade and were procured from Shanghai Macklin Biochemical Technology Co., Ltd. (Shanghai, China).

### 2.2. Pretreatment of SSG

The method described by Liu et al. [18] was adapted for SSG pretreatment with some modifications. The Pacific white shrimp (*Litopenaeus vannamei*) meat was treated with deionized water and homogenized using a chopper (MQ 5025, Braun, Kronberg im Taunus, Germany) to achieve a consistent mince. The minced shrimp was blended with NaCl (0.5%, *w*/*w*) for 2 min. Different amino acids (L-Arg, L-Lys, L-Pro) were introduced into minced shrimp at a concentration of 1%, which was determined to be optimal based on preliminary experiments (Figure S1). The minced shrimp was centrifuged for 5 min at 5000× *g* using a micro-benchtop centrifuge (H1650, Xiangyi Instrument Co., Hunan, China) to remove air bubbles. The samples were first held at 40 °C for 30 min (core gel temperature: 38.6 °C) and immediately heated at 90 °C for another 30 min (core gel temperature: 87.3 °C), with subsequent rapid cooling in an ice bath at an average rate of approximately 3–4 °C/min, and stored at 4 °C. Based on a slightly modified method from Liu et al. [18], the SSG formulated with 0.5% NaCl and 3% NaCl were labeled as the low-salt control (LC) group and high-salt control (HC) group, respectively.

### 2.3. Gel Strength

Gel strength was assessed employing a texture analyzer (TA-XT Plus, Stable Micro System, Surrey, UK) [19]. Cylindrical specimens (20 mm diameter × 10 mm height) were compressed with a P/0.5 probe at pre-test, test, and post-test speeds of 1.5, 1.0, and 1.0 mm/s, respectively, under 40% deformation and a 5.0 g trigger force. The gel’s breaking force and deformation were determined by separately measuring the peak force at the gel’s breaking point and the probe displacement. Gel strength values were computed according to Equation (1):

(1) Gel strength (g·cm) = breaking force (g) × breaking deformation (cm)

### 2.4. Textural Properties

Texture attributes of SSG were assessed via a texture analyzer (TA-XT Plus, Stable Micro System, Surrey, UK) [20]. Cylindrical specimens (20 mm diameter × 10 mm height) were analyzed in TPA mode employing a P/50 probe. Pre-test, test, and post-test velocities were established at 1.5, 1.0, and 1.0 mm/s, respectively. Compression degree was configured to 40%, with trigger force adjusted to 5.0 g. Evaluated parameters encompassed hardness, springiness, cohesiveness, and chewiness.

### 2.5. Cooking Loss

Cooking loss of samples was carried out as the method described by Man et al. [21]. The mass of minced shrimp was recorded as W_1_. Following a two-stage water-bath heating process (40 °C for 30 min, then 90 °C for 30 min) and cooled to 4 °C, the surface water was wiped off, and the mass of SSG was registered as W_2_, with the cooking loss computed according to the subsequent equation (Equation (2)):

(2) Cooking loss (%) = (W_1_ − W_2_/W_1_) × 100

where W_1_ and W_2_ corresponded to the SSG mass prior to and following thermal processing in the water bath, respectively.

### 2.6. Water-Holding Capacity (WHC)

The WHC was assessed using the modified protocol of Zhang et al. [19]. Approximately 1 g sample (m_1_) was centrifuged at 10,000× *g* for 15 min at 4 °C. The mass after centrifugation was registered as m_2_, and WHC was computed via Equation (3):

(3) WHC (%) = m_2_/m_1_ × 100

where m_1_ and m_2_ were designated as the pre-centrifugation and post-centrifugation masses, respectively.

### 2.7. Water Distribution

The low-field nuclear magnetic resonance (LF-NMR) technique (MesoMR23-060V-1, Niumag Analytical Instrument Co., Ltd., Shanghai, China) was employed to determine water migration in SSG, based on a slightly modified method from Shang et al. [20]. Cylindrical specimens (20 mm diameter × 10 mm height) were placed into a 40 mm tube to determine *T*_2_ relaxation times using a multi-pulse echo sequence (CPMG). Measurement parameters encompassed a recovery delay of 3500 ms, 0.5 ms echo time, and 10,000 echo count.

### 2.8. Magnetic Resonance Imaging (MRI)

The MRI analysis of gels was conducted following the protocol established by Man et al. [21], with minor adjustments using an NMR technique (MesoMR23-060V-1, Niumag Analytical Instrument Co., Ltd., Shanghai, China). Specimens were arranged in a tube with a 40 mm diameter. The sample was segmented into two layers, each 1.0 mm thick, with a 1.0 mm gap between them. The parameters were TR = 2000 ms, TE = 20 ms.

### 2.9. Chemical Forces

The chemical forces were assessed following Zhang et al. [22] with slight alterations. Initially, 5 g SSG was thoroughly dispersed in 25 mL of Sa (0.6 M NaCl) and processed (homogenization: 6000 rpm, 2 min; shaking: 180 rpm, 1 h; centrifugation: 10,000× *g*, 20 min, 4 °C). The pellet was successively extracted with Sb (1.5 M urea, 0.6 M NaCl), Sc (8 M urea, 0.6 M NaCl; twice), and Sd (0.5 M β-mercaptoethanol, 0.6 M NaCl, 8 M urea, pH 7.0) under identical conditions. The final insoluble residue was dissolved in 1 M NaOH. The protein content in each supernatant corresponded to ionic bonds (Sa), hydrogen bonds (Sb), hydrophobic interactions (Sc), and disulfide bonds (Sd), respectively.

### 2.10. Fourier Transform Infrared Spectroscopy (FT-IR)

Following the method previously reported by Zhang et al. [23], the protein structures of SSG were identified using the FT-IR spectrometer (Spectrum Two, PerkinElmer Co., Waltham, MA, USA). The samples were freeze-dried, then mixed with potassium bromide at a proportion of 1:100 (*w*/*w*), and fully ground under a deuterium lamp. Spectra were acquired in the 500–4000 cm^−1^.

### 2.11. Raman Spectroscopy

The Raman spectra of SSG were measured by a Raman microscope ( LabRAM HR Evolution, Horiba Scientific, Ltd., Kyoto, Japan) based on the method of Song et al. [24]. Spectra were acquired in the 500–3500 cm^−1^ range with a 532 nm excitation laser. The experimental parameters were set as follows: three scans, a 30 s exposure time, a resolution of 2 cm^−1^, a sampling speed of 120 cm^−1^/min, and data collection at 1 cm^−1^ intervals.

### 2.12. Microstructure

A cryo-scanning electron microscope (Cryo-SEM, SU8000, Hitachi Co., Ltd., Tokyo, Japan) was employed for microstructural analysis of SSG [23]. Sample preparation involved mounting on a copper stub, rapid freezing in liquid nitrogen, and sublimation at −70 °C for 40 min. After platinum coating (10 mV, 60 s), microstructural observation was conducted at 10.0 kV with 5000× and 10,000× magnification.

### 2.13. In Vitro Simulated Digestibility

The in vitro digestion procedure for the SSG was adapted from the standardized INFOGEST protocol with specific modifications following Lee et al. [25]. In brief, each sample (5.0 g, approximately 1.7 cm × 1.7 cm × 1.7 cm) was first homogenized at 6000 rpm for 10 s with 5 mL of simulated salivary fluid (pH 7) and subjected to a 5 min incubation under shaking (150 rpm) at 37 °C. The gastric phase was initiated by adding 10 mL of simulated gastric fluid (pH 3) containing pepsin (2000 U/mL), followed by a 2 h incubation under identical shaking and temperature conditions. Subsequently, the intestinal digestion was carried out by introducing 20 mL of simulated intestinal fluid (pH 7) supplemented with pancreatin (100 U/mL of trypsin activity) and bile (10 mM), with further incubation for 2 h. The reaction was halted through a 5 min boiling step. Immediately after, centrifugation was performed at 10,000× *g* for 10 min. The resulting pellet was oven-dried at 50 °C to determine the residual insoluble protein. The digestibility was calculated using the following equation (Equation (4)):

(4) Digestibility (%) = (M_1_ − M_2_/M_1_) × 100

where M_1_ and M_2_ represented the dry matter content of the samples before and after digestion, respectively.

### 2.14. Extraction of Myofibrillar Proteins

Myofibrillar proteins were extracted following the procedure of Li et al. [26] with minor alterations. Litopenaeus vannamei was decapitated, shelled, and deveined, then minced for 2 min using a chopper (MQ 5025 plus, Braun, Kronberg im Taunus, Germany). The minced shrimp was blended at a ratio of 1:6 (*w*/*v*) with low-salt buffer (0.05 M NaCl, 20 mM Tris-HCl, 2 mM MgCl2, 1 mM EGTA, pH 7.0) and homogenized at 6000 rpm for 3 min. The sample was pelleted by centrifugation (10,000× *g*, 15 min, 4 °C) (H1650, Xiangyi Instrument Co., Hunan, China). The liquid portion was removed, and this isolation process was iterated three times to remove sarcoplasmic proteins. The obtained pellet was homogenized in high-salt buffer (0.6 M NaCl, 20 mM Tris-HCl, pH 7.0) at a 1:10 ratio (*w*/*v*) for 3 min. The mixture was extracted at 4 °C for 12 h, followed by centrifugation at 10,000× *g* for 30 min at 4 °C. The liquid portion was filtered through four layers of gauze and dialyzed. The final myofibrillar protein solution was obtained for subsequent experiments. The procedure resulted in myofibrillar proteins of 5 mg/mL and an extraction efficiency of about 10%.

### 2.15. Determination of pH of Myofibrillar Proteins

Myofibrillar proteins from Pacific white shrimp were diluted to 1 mg/mL and distributed into five treatments: a LC group containing 0.5% (*w*/*v*) NaCl; a HC group containing 3% (*w*/*v*) NaCl; and three amino acid groups (L-Arg, L-Lys, L-Pro), each supplemented with 1% (*w*/*v*) of the respective amino acid and 0.5% (*w*/*v*) NaCl. The pH of each group was determined with a pH meter (PHS-3E, INASE Scientific Instruments Co. Ltd., Shanghai, China)

### 2.16. Determination of Turbidity of Myofibrillar Proteins

Turbidity measurement was conducted by referring to Li et al. [27]. The absorbance at 660 nm of the myofibrillar protein solution was measured using a multi-functional microplate detection analyzer (Synergy H1, Agilent Technologies Inc., Shanghai, China), which was recorded as turbidity to reflect the aggregation state of the proteins.

### 2.17. Determination of Solubility of Myofibrillar Proteins

Protein solubility was evaluated according to Man et al. [21]. The MP solution was subjected to centrifugation (8500 rpm, 10 min, 4 °C). The protein content before centrifugation (denoted as C_0_) and that of the supernatant after centrifugation (denoted as C_1_) were determined using the Bradford method. Protein solubility was derived using Equation (5) below:

(5) Protein solubility (%) = C_1_/C_0_ × 100

### 2.18. Molecular Docking of L-Arg, L-Lys, and L-Pro with Myosin from Pacific White Shrimp (Litopenaeus vannamei)

Myosin was chosen as the representative target because it constitutes 55–60% of myofibrillar protein and is the primary cross-linking substrate in surimi gels [28]. The *Litopenaeus vannamei* myosin sequence (ID: R0T75475.1) was retrieved from the National Center for Biotechnology Information database (https://www.ncbi.nlm.nih.gov/) to perform homology modeling, since no crystal structure existed in the RCSB Protein Data Bank database (https://www.rcsb.org/). The modeling template was selected through a comprehensive evaluation of sequence homology and similarity. The three-dimensional structures of L-Arg, L-Lys, and L-Pro were sourced from the PubChem database (https://pubchem.ncbi.nlm.nih.gov/). We employed the SWISS-MODEL online platform (https://swissmodel.expasy.org/) to perform homology modeling. Molecular docking was performed using Smina (a derivative of AutoDock Vina 1.1.2) with rigid protein side chains. The predicted binding site of the protein was defined as the center of a 40 Å × 40 Å × 40 Å search space. The center of this box was set to the coordinates x = −18.629, y = −5.119, z = −4.829. Molecular docking analysis and visualization were performed using PyMOL 2.6.2 and Discovery Studio software 4.5.

### 2.19. Data Analysis

All experimental procedures were conducted in triplicate. The graphical representation of the data was produced via Origin Pro 2021 (Origin Lab Corporation, Northampton, MA, USA). Statistical evaluation was executed with SPSS 25.0 software (IBM SPSS Statistics, Chicago, IL, USA). A *p* < 0.05 was considered to denote statistical significance. Values are reported as mean ± standard deviation. Statistical analysis was performed using analysis of variance (ANOVA), and the normality assumption was verified.

## 3. Results and Discussion

### 3.1. Appearance of Low-Salt SSG with Various Amino Acids

**Figure 1:**
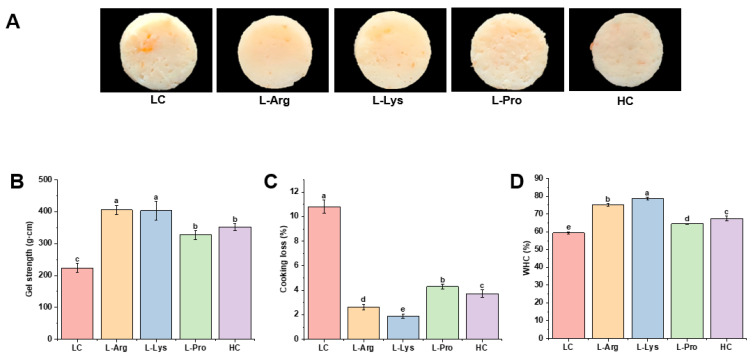
Effects of different amino acids on the appearance (**A**), gel strength (**B**), cooking loss (**C**), and WHC (**D**) of low-salt SSG. Different lowercase letters in the same index denote significant differences (*p* < 0.05).

The macroscopic pictures of SSG directly correlate with their product characteristics. The apparent attributes of samples with various amino acids are depicted in Figure 1A. The LC group displayed large pores with heterogeneous distribution on the surface. Compared with the LC group, the HC group had a smoother surface and more uniform pore sizes. Supplementation with L-Arg, L-Lys, and L-Pro effectively reduced the pore sizes of low-salt SSG, resulting in a more compact structure. Among these, the L-Arg and L-Lys groups stood out, with surfaces that were exceptionally smoother and more homogeneous, outperforming the HC group.

### 3.2. Gel Strength and TPA of Low-Salt SSG with Various Amino Acids

Gel strength constitutes a key parameter for assessing gelation properties of SSG, where an increased value indicates a more stable internal network [29]. The breaking force and deformation reflect the hardness and elasticity of SSG, respectively [30]. As shown in Figure S2, compared to the LC group, supplementing low-salt SSG with the three amino acids did not alter breaking deformation but significantly increased breaking force. Gel strength is a comprehensive measure of the relationship between breaking force and deformation. As shown in Figure 1B, the addition of all amino acids significantly increased the gel strength of samples, especially L-Arg (405.33 g·cm), and L-Lys (403.44 g·cm), showing superior effects compared to the HC group (351.90 g·cm), which was consistent with the improved macroscopic performance (Figure 1A). This was because alkaline amino acids might promote protein dissolution, and the positively charged side chains (such as the guanidinium group in L-Arg [31] and the ε-amino group in L-Lys [32]) might facilitate the exposure of reactive groups, which potentially enhanced intermolecular cross-linking and resulted in a gel network with enhanced structural density and uniformity in three dimensions, leading to a significant increase in breaking force and gel strength, as supported by Wang et al. [33]. Compared to the LC group (223.99 g·cm), the gel strength was elevated to 326.94 g·cm in the L-Pro group. This effect was likely due to the aided solubilization induced by L-Pro [16], leading to the establishment of a more stable gel network.

TPA is used to simulate the process of chewing gels, which provides a direct indication of the sensory qualities of SSG [34]. Table 1 presents the hardness, springiness, cohesiveness, and chewiness of low-salt SSG supplemented with various amino acids. The incorporation of various amino acids into SSG increased hardness to varying degrees. The hardness of samples with L-Lys, L-Arg, and L-Pro was 1.47-, 1.35-, and 1.18-fold higher than that of the LC group, respectively, and L-Lys and L-Arg groups surpassed the values for the HC group. L-Arg might be able to bind to hydrophobic residues of myofibrillar proteins [35], and L-Lys might alter the conformation and charge distribution of myofibrillar proteins and bind to the aromatic and acidic groups [36]. These interactions possibly enhanced the development of a three-dimensional gel framework in low-salt SSG, which significantly enhanced gel hardness [37]. Compared to the LC group, the changes in hardness in the L-Pro group might be attributed to the promotion of protein interactions due to the cyclic structure in L-Pro [38]. Relative to the LC group, L-Arg and L-Lys groups markedly enhanced cohesiveness and chewiness, achieving comparable or even superior effects to the HC group. The improvement might be attributed to the solubilizing effect of alkaline amino acids on myofibrillar proteins [21]. L-Pro has a cyclic structure [38] and might integrate into the myofibrillar-protein-based protein networks without significantly altering the overall structure of the proteins, resulting in cohesiveness and chewiness remaining relatively stable relative to the LC group. Springiness represents a pivotal metric for quantifying the degree of myofibrillar protein cross-linking [32]. Table 1 demonstrates that L-Arg and L-Lys supplementation markedly improved springiness relative to the LC group. The underlying mechanism was presumably that these amino acids facilitated a well-ordered arrangement. The springiness of samples containing L-Pro remained comparable to that of the LC group. Moreover, Man et al. [21] demonstrated that 0.6% L-Arg and L-Lys supplementation significantly improved the springiness of low-salt mixed SSG. Therefore, alkaline amino acids significantly improved the gel strength, hardness, cohesiveness, and chewiness of the samples, surpassing those of the HC group. The four key texture parameters—hardness, springiness, cohesiveness, and chewiness—collectively defined the desirable sensory attributes of low-salt SSG, contributing to a satisfying bite, resilient mouthfeel, structural integrity, and enhanced juiciness [39]. These findings confirm that the type of exogenous amino acids could significantly affect gel strength and textural properties, and enhance the sensory experience of consumers.

### 3.3. Cooking Loss and WHC of Low-Salt SSG with Various Amino Acids

Cooking loss refers to the release of liquids and small amounts of soluble substances from the gel matrix during thermal processing [36]. As shown in Figure 1C, the cooking loss was significantly decreased from 10.80% (LC group) to 1.89% (L-Lys group), 2.63% (L-Arg group), and 4.30% (L-Pro group), respectively. This reduction was primarily attributed to these amino acids promoting protein cross-linking, thereby reinforcing the protein network of the gels [40]. This enhanced network effectively minimized water loss during cooking, leading to a significant reduction in cooking loss. In particular, L-Lys and L-Arg exhibited lower cooking loss than the HC group, as they effectively prevented excessive protein aggregation and reinforced the gel matrix [36], thereby diminishing moisture expulsion and the loss of solubilized components during heat treatment. According to references, while supplementing 1–3% L-Arg or L-histidine exerted no significant effect on cooking loss in high-salt *Amur sturgeon* surimi gel systems, both amino acids proved effective in reducing it under low-salt conditions [41], which aligned with our results.

WHC refers to the gel’s capability to immobilize water within its three-dimensional network through protein–water interactions [42]. The WHC is primarily determined by the structural integrity of the gel network, with denser structures demonstrating improved water-binding properties [3]. Figure 1D shows that all treatments increased WHC to varying degrees over the LC group, while L-Pro addition improved WHC but not to the level of the HC group. Consistent with the results shown in Figure 1A, smaller pores indicated a smoother and more compact surface of SSG, which was positively correlated with its WHC. The incorporation of L-Pro might enhance the solubility of myofibrillar proteins [16], thus facilitating greater involvement of proteinaceous components in gel framework development and elevating water-binding capacity. Remarkably, the WHC observed in L-Arg and L-Lys groups surpassed the performance of the HC group, with increases of 26.79% and 32.74% compared to the LC group, respectively. This effect was likely mediated by the ability of alkaline amino acids to suppress protein aggregation, thereby facilitating the development of hydrolocked gel structures [36]. Man et al. [21] further demonstrated that low-salt mixed SSG reached an optimal state in the presence of alkaline amino acids (adding 0.3% L-Lys resulted in a value 1.1 times that of the HC group), which promoted the development of a more compact gel architecture, substantially improving WHC and markedly reducing cooking loss. Moreover, extensive references have demonstrated that adding exogenous additives could effectively enhance WHC of low-salt surimi systems. Compared to the control group, high-pressure processing pretreatment increased the WHC of silver carp surimi gel containing κ-carrageenan by 1.1-fold [43]. In low-salt systems, L-histidine enhanced WHC of reduced-salt surimi gel by approximately 1.1-fold relative to the LC group [44]. Meanwhile, supplementation with L-Arg led to a 1.2-fold increase in WHC of low-salt Chinese SSG relative to the LC group [18]. In our study, the sample with L-Lys exhibited the best performance, with respective increases of 1.3 times and 1.2 times over the LC and HC groups. In conclusion, the above results proved that L-Lys and L-Arg enhanced the binding ability of protein to water, showing the lowest cooking loss and the highest WHC. The obtained data underscore that amino acid type is a pivotal factor in regulating moisture-related attributes.

### 3.4. Water Distribution and Water State of Low-Salt SSG with Various Amino Acids

**Figure 2:**
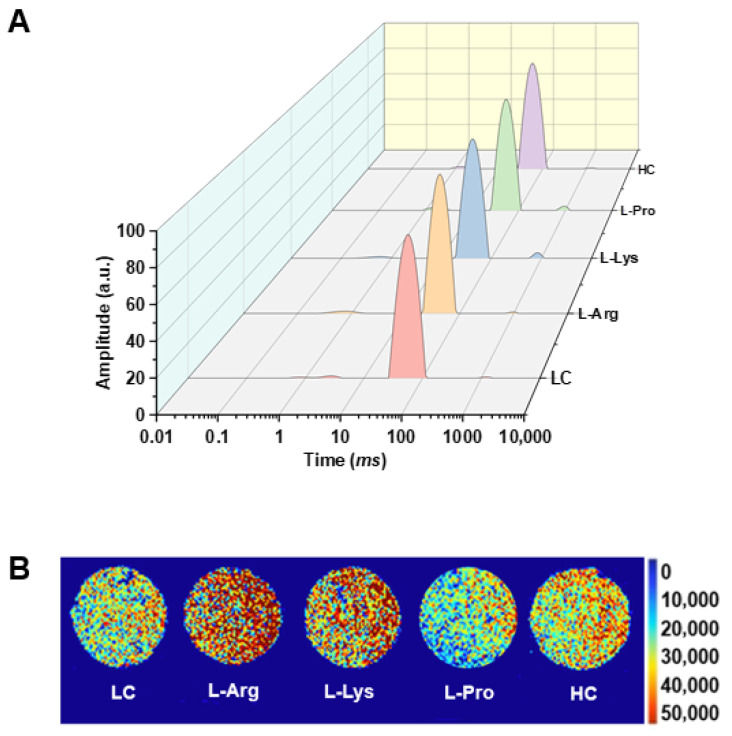
Effects of different amino acids on water migration (**A**) and water distribution (**B**) of low-salt SSG.

LF-NMR enables non-destructive measurement of water molecule mobility and distribution in gel systems, providing precise characterization of water states [45]. Figure 2A shows water relaxation profiles in low-salt SSG with various amino acids, and the *T*_2_ relaxation times reveal three distinct peaks: bound water *T*_21_ (0–10 ms), immobilized water *T*_22_ (10–300 ms), and free water *T*_23_ (300–3000 ms) [46]. Table 2 shows the water status of low-salt SSG supplemented with various amino acids. In comparison to the LC group, supplementation with alkaline amino acids markedly diminished *T*_21_ and *T*_22_ relaxation times, indicating that bound water and immobilized water were stabilized and tightly bound within the gel matrix, which was macroscopically reflected in the smoother surface observed in Figure 1A. This is supported by previous studies that show that L-Arg and L-Lys reinforce hydrogen bonds in gel networks [47]. These amino acids might associate with myofibrillar proteins via hydrogen bonding [48], thereby augmenting the moisture retention capability of samples. L-Pro supplementation exhibited no significant variation in *T*_21_ and *T*_22_ parameters (*p* > 0.05) relative to the LC group (Table 2). Although L-Pro might enhance myofibrillar protein interactions to promote aggregate formation and modestly strengthen water binding [16], the effect was too weak to induce detectable changes in relaxation times. All amino acid groups (L-Arg, L-Lys, L-Pro) showed significantly shortened *T*_23_ compared with the LC group. This observation was likely due to the denser gel network, which retained more water and reduced its mobility. This reduction might result from strengthened interactions between these amino acids and myofibrillar proteins, which restricted the diffusion of free water and promoted a homogeneous and dense spatial framework, thereby improving textural properties (Table 1).

The signal intensity in MRI is typically proportional to water content, with red and blue indicating a high and low hydrogen proton density within the gel matrix, respectively [49]. Figure 2B reveals that L-Lys and L-Arg treatments significantly expanded high-proton-density regions compared to the LC group, indicating enhanced water-binding capacity that effectively restricted water loss [21], correlated with the improved WHC (Figure 1D) and reduced cooking loss (Figure 1C). Man et al. [21] further confirmed that L-Lys and L-Arg groups elevated the hydrogen proton density above the HC group. However, the pseudo-color images of SSG with the L-Pro group exhibited less distinct differences, probably since the effect of L-Pro was insufficient to significantly affect MRI results.

### 3.5. Chemical Forces of Low-Salt SSG with Various Amino Acids

**Figure 3:**
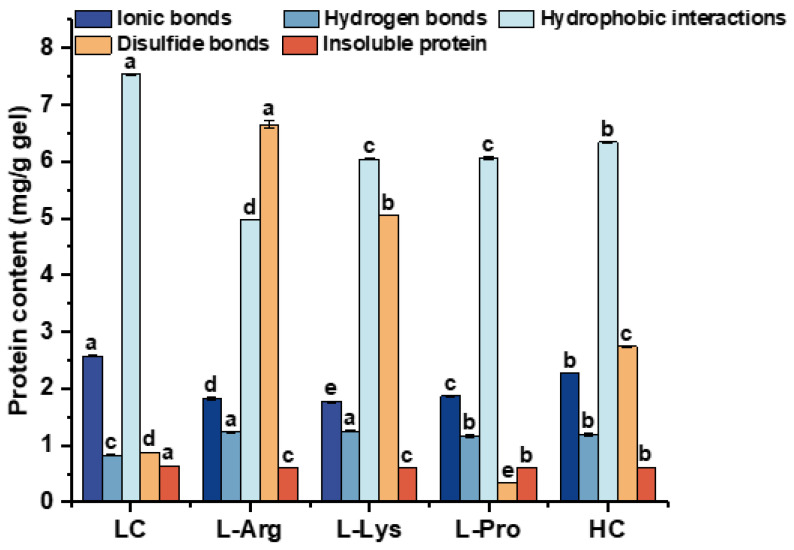
Effects of different amino acids on chemical forces of low-salt SSG. Different lowercase letters in the same index denote significant differences (*p* < 0.05).

The chemical forces that form and support the network structure of SSG include ionic bonds, hydrogen bonds, hydrophobic interactions, and disulfide bonds, which also determine the properties of gels [50]. When a gel was treated with chemical reagents designed to disrupt specific bonds, the effects of interactions on gel formation could be assessed by measuring the soluble protein release. This approach has been widely used to analyze the chemical forces in protein gels or surimi products. However, the chemical reagents employed lack absolute specificity for a single type of molecular interaction, which might produce certain effects and thus have certain limitations [51]. The results of chemical forces of low-salt SSG with various amino acids are shown in Figure 3. The amount of insoluble protein could correlate with the degree of non-disulfide covalent cross-linking [52]. The supplementation of L-Arg and L-Lys elevated disulfide bonds in low-salt SSG, potentially reflecting enhanced overall covalent cross-linking, which was consistent with their superior gel strength (Figure 1B). The potential mechanism might be due to amino acid residue specificity: the guanidinium group of L-Arg, characterized by its unique double bond structure, might abstract hydrogen atoms from sulfhydryl (SH) groups, thus potentially facilitating disulfide bond formation [18]. L-Lys might expose the free SH group of myofibrillar proteins through electrostatic repulsion by the ε-amino group, which potentially enhances the cross-linking of protein disulfide bonds [53]. In contrast, L-Pro significantly decreased disulfide bonds, suggesting that its cyclic side chain promoted non-disulfide covalent linkages while sterically hindering disulfide bond formation [38].

All amino acid groups (L-Arg, L-Lys, and L-Pro) showed significantly reduced hydrophobic interactions, ionic bonds and insoluble protein content compared to the LC group, which was probably due to the side chain groups of amino acids interacting with neighboring molecules, masking the exposure of hydrophobic groups [31]. Alkaline amino acids might disrupt intramolecular ionic bonds via electrostatic attraction between their cationic side chains and anionic residues in myofibrillar proteins [54]. Previous studies have shown that L-Pro residues could modulate the helical structure by introducing kinks between segments [38], which might alter local conformation and weaken electrostatic interactions, ultimately reducing the number of ionic bonds. Compared to the LC group, the L-Arg, L-Lys, and L-Pro groups significantly decreased insoluble protein content. These amino acids might suppress the formation of insoluble aggregates and promote cross-linking of soluble proteins into a cohesive gel network.

Incorporation of L-Arg, L-Lys, and L-Pro augmented hydrogen bond content in samples versus the LC group, likely resulting from preferential H-bond formation between the amino moiety of L-Arg and carbonyl oxygen functionalities within myofibrillar protein chains [55]. In addition, the lone-pair-electron amino groups in Lys also potentially form hydrogen bonds with the carbonyl oxygen atoms of myofibrillar proteins [36]. The cyclic ring structure of L-Pro might induce local conformational rearrangements that expose adjacent buried polar sites [16], which might form additional hydrogen bonds with surrounding water molecules or amino acid side chains, leading to a significant increase in hydrogen bond content.

### 3.6. FT-IR Spectra of Low-Salt SSG with Various Amino Acids

**Figure 4:**
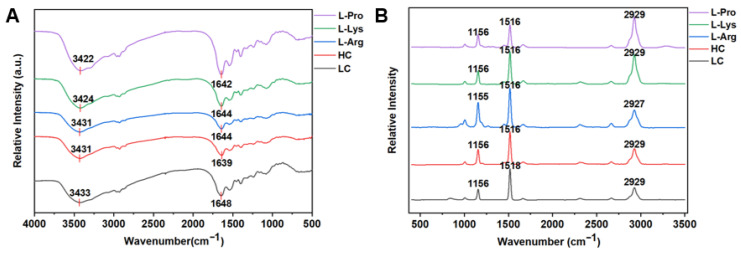
Effects of different amino acids on FT-IR spectra (**A**) and Raman spectra (**B**) of low-salt SSG.

FT-IR is an effective technique for analyzing changes in protein functional groups and microenvironment [56]. As illustrated in Figure 4A, the FT-IR spectra of low-salt SSG containing various amino acids revealed that no novel covalent bonds were induced in myofibrillar proteins. The characteristic absorption peak around 3000–3500 cm^−1^ is usually associated with the O-H stretching vibration [57]. Compared to the LC group (3433 cm^−1^), the L-Arg, L-Lys, and L-Pro groups exhibited shifts to lower wavenumbers, specifically to 3431 cm^−1^, 3424 cm^−1^, and 3422 cm^−1^, respectively, indicating that the addition of amino acids promoted hydrogen bond formation, consistent with the increased hydrogen bond content observed in Figure 3. L-Arg and L-Lys molecules contained hydrogen bond donors, specifically the guanidinium group in L-Arg [31] and the ε-amino group in L-Lys [32], respectively. These groups could form additional hydrogen bonds, thereby strengthening intra- or intermolecular hydrogen bond networks involving proteins and water molecules [58]. L-Pro had a cyclic side chain attached to the α-carbon, which might alter the spatial conformation of the protein molecule [38], exposing more polar sites and thereby forming a hydrogen-bond network with surrounding water molecules or protein molecules. In addition, the amide I band (1600–1700 cm^−1^) serves as the primary basis for characterizing the secondary structure of proteins [59]. Quantitative analysis of secondary structure revealed that alkaline amino acids induced a significant change in protein conformation from a relatively disordered state to a highly ordered one [59]. This transition was primarily characterized by a marked increase in the combined content of regular structures (α-helix and β-sheet) from approximately 77% to over 97%, concomitant with a sharp decrease in the proportion of β-turn from about 23% to below 3% compared to the LC group (Table 3). These findings further supported the argument that positively charged alkaline amino acids could promote protein unfolding and induce conformational changes. This process led to increased exposure of SH groups, thereby resulting in a greater number of disulfide bonds (Figure 3). Additionally, the more ordered secondary structures of myofibrillar proteins might effectively facilitate the formation of hydrogen bond networks within or between protein molecules. In contrast, L-Pro did not alter the structure of myofibrillar proteins. All amino acid treatments induced a shift to approximately 1639 cm^−1^–1644 cm^−1^ (vs. 1648 cm^−1^ for the LC group), suggesting stronger electrostatic interactions and a more ordered secondary structure [23]. Qian et al. [59] showed that changing the concentration and type of salt in *Penaeus vannamei* shrimp surimi did not induce novel covalent bonds. Shi et al. [60] also demonstrated that supplementation with L-Arg and TGase enhanced the O-H vibrational intensity of water molecules.

### 3.7. Raman Spectra of Low-Salt SSG with Various Amino Acids

Raman spectra is a powerful tool for characterizing spatial conformational changes in proteins [61]. Figure 4B shows the Raman spectra of SSG prepared with different amino acids. Changing the amino acid type did not generate new covalent bonds. The spectral region between 2800 cm^−1^ and 3100 cm^−1^ in Raman spectra corresponds to C–H stretching vibrations [62]. Compared to the LC group, supplementation with L-Arg, L-Lys, and L-Pro increased the intensity of the C–H stretching vibration bands, suggesting that all three amino acids altered the microenvironment of proteins [63]. The Raman peaks at 830 cm^−1^ and 850 cm^−1^ originate from vibrational modes of the para-substituted phenyl ring of tyrosine residues. The intensity ratio (I_850_/I_830_) is sensitive to the microenvironment of the tyrosine and the hydrogen-bonding status of the phenolic hydroxyl group [63]. As shown in Table 4, the I_850_/I_830_ values showed no significant difference in low-salt SSG treated with L-Arg and L-Pro compared to the LC group (*p* > 0.05), indicating that these residues did not appreciably alter the microenvironment of tyrosine. Compared to the LC group, the lower I_850_/I_830_ in the L-Lys group indicated that tyrosine residues were likely located in the protein interior or acted as strong hydrogen bond donors [63], and the protonated ε-amino group acted as a hydrogen bond acceptor [32].

The I_760_/I_1003_ ratio, an indicator of tryptophan (Trp) exposure, decreased significantly from 0.45 in the LC group to 0.27 and 0.36 with alkaline amino acids, respectively (Table 4). This reduction reflected the increased exposure of Trp residues and a more polar microenvironment, which likely facilitated the exposure of other buried internal groups such as SH groups [64].

### 3.8. Microstructure of Low-Salt SSG with Various Amino Acids

**Figure 5:**
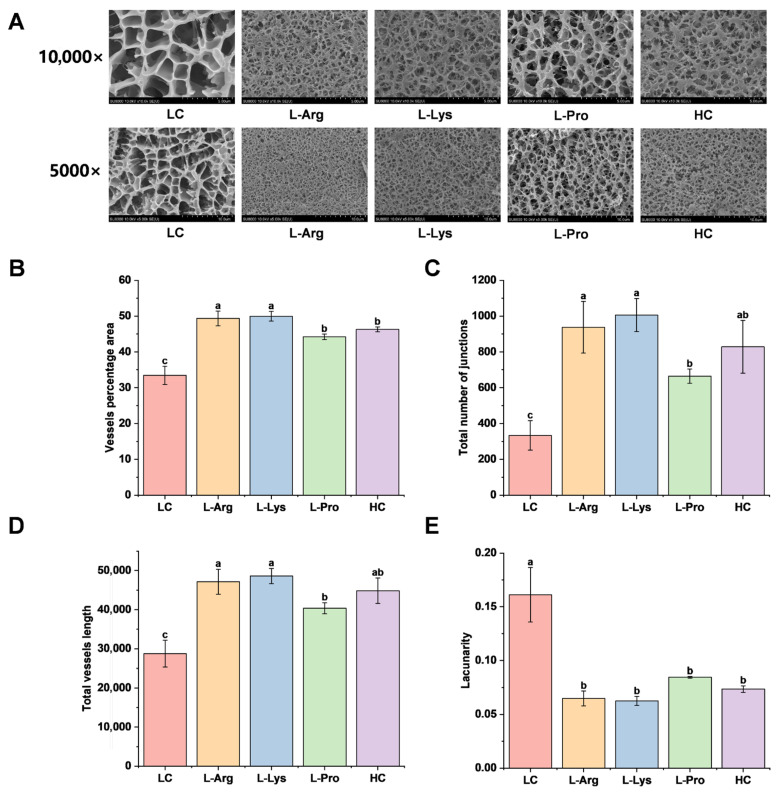
Effects of different amino acids on microstructural properties of low-salt SSG. The images were captured using a magnification of 10,000× and 5000× (**A**), vessel percentage area (**B**), total number of junctions (**C**), total vessel length (**D**), and lacunarity (**E**). Different lowercase letters in the same index denote significant differences (*p* < 0.05).

The microstructures of SSG with various amino acids were analyzed by Cryo-SEM. As illustrated in Figure 5A, the microscopic pores of the LC group were relatively large, showing a loose gel network with unevenly distributed cavities, while the microscopic network structure of the HC group was denser and more organized, which might be ascribed to enhanced solubilization of myofibrillar proteins at elevated salt concentrations, leading to a more uniform gel matrix [33]. The microstructure and pore homogeneity of low-salt SSG supplemented with L-Pro were superior to those of the LC group, but inferior to the HC group, suggesting that L-Pro could promote protein interactions [16], consequently generating a comparatively dense framework. As shown in Figure 5B–E, the quantitative network analysis was performed to further examine the microstructure of gels following the addition of amino acids. The key parameters showed substantial increases; for example, the vessel percentage area increased by 32.2% to 34.5%, the total number of junctions rose by 99.0% to 201.3%, and the total vessel length grew by to 40.5% to 69.2%, respectively. In contrast, lacunarity significantly decreased by 46.9% to 60.6%. Therefore, this denser network structure physically restricted the mobility of water molecules, leading to a corresponding decrease in *T*_2_ relaxation times (Table 2). Moreover, the incorporation of alkaline amino acids yielded more organized and compact networks that exceeded the HC group. This phenomenon presumably arose from enhanced S–S bridge formation and the masking of hydrophobic groups (Figure 3), which facilitated myofibrillar protein unfolding and the unmasking of supplementary binding sites [65], giving rise to a tightly cross-linked spatial gel matrix. Thus, L-Arg and L-Lys enabled a superior three-dimensional network density in SSG, and the microstructures were consistent with the macroscopic results (Figure 1A).

### 3.9. Simulated In Vitro Digestibility of Low-Salt SSG with Various Amino Acids

**Figure 6:**
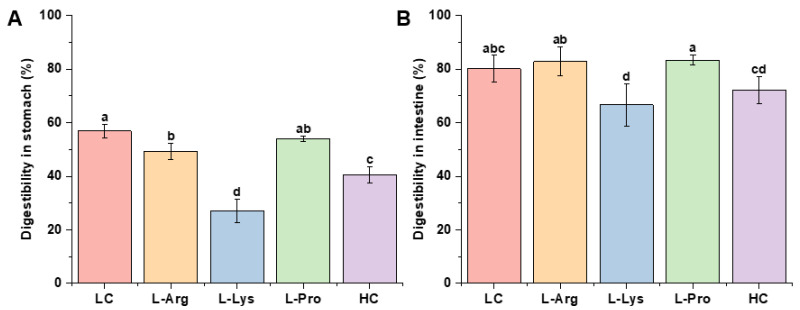
Effects of different amino acids on digestibility in stomach (**A**) and intestine (**B**) of low-salt SSG. Different lowercase letters in the same index denote significant differences (*p* < 0.05).

In vitro digestibility is a key metric of the nutritional value of SSG, and it is governed by the gel matrix composition, structure, and surface properties that jointly modulate gastric and intestinal digestion [17]. Figure 6A,B present the in vitro digestion profiles of SSG in the gastric and intestinal phases. During the gastric phase, the LC group exhibited the highest digestibility (56.81%). The supplementation of L-Lys and L-Arg significantly reduced the digestibility of samples to 27.14% and 49.28%, respectively. There was no significant effect observed with the addition of L-Pro. The decreased digestibility with L-Lys and L-Arg might form a more compact three-dimensional network structure, as evidenced by their significant increase in gel strength (Figure 1B) and high WHC (Figure 1D). Generally, a higher cross-linking density and a more compact structure might act as a barrier during the initial gastric phase, impeding the diffusion and penetration of pepsin and ultimately reducing digestibility [66]. Although the cyclic structure of L-Pro could alter protein conformation [38], potentially modifying protein aggregation states and gel networks, the effects did not reach the threshold to influence enzyme permeability and activity, resulting in no significant difference in digestibility. However, during the intestinal phase, only the L-Lys group exhibited a significantly lower digestibility than the LC group (*p* < 0.05). The densification of gel structure resulting from reduced porosity might limit the diffusion of digestive proteases (such as pepsin and trypsin) into the gel interior and potentially decrease the surface area available for effective contact between enzyme molecules and substrate proteins. Another hypothesis suggested this sustained suppression might arise from the unique ε-amino group of L-Lys, forming a highly stable network, potentially via covalent cross-linking such as the ε-(γ-glutamyl) lysine bonds [67]. This stable network would effectively resist degradation by pancreatic proteases, thereby delaying enzymatic hydrolysis in the intestine. In contrast, the SSG formed with the other amino acids was likely more susceptible to disruption in the intestinal environment, facilitating efficient enzymatic hydrolysis and resulting in digestibility comparable to the LC group. Moreover, according to the references, the digestive properties and nutrient release patterns of Pacific white SSG were highly dependent on their cross-linked network structure, which ultimately influenced the absorption efficiency of these digestion end-products in the human body [68]. In summary, the addition of L-Arg reduced the gastric digestibility but had no significant impact on intestinal digestibility. Conversely, L-Lys enhanced gel stability by both limiting the diffusion of digestive proteases and promoting the formation of ε-(γ-glutamyl) lysine bonds, leading to significantly lower digestibility in both the gastric and intestinal phases. These findings highlight that amino acid type could modulate surimi gel digestibility.

### 3.10. pH Value of Myofibrillar Proteins Solution with Various Amino Acids

**Figure 7:**
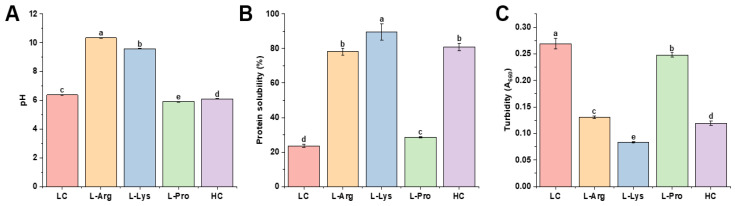
Effects of different amino acids on pH (**A**), protein solubility (**B**), and turbidity (**C**) of low-salt shrimp myofibrillar proteins. Different lowercase letters in the same index denote significant differences (*p* < 0.05).

The pH value is a critical parameter for understanding the amino-acid-mediated regulation of surimi gel properties, as it directly modulates the surface charge of myofibrillar proteins. Figure 7A shows the pH values of myofibrillar proteins with various amino acids. Relative to the LC group, the incorporation of L-Arg and L-Lys markedly enhanced the pH values to 10.35 and 9.59, respectively. L-Arg’s guanidinium group [31] and L-Lys’s ε-amino group [32] act as proton-accepting groups, binding free H^+^ in the solution and raising pH. Consistent with Sun et al. [69], higher pH enhances myofibrillar proteins’ hydration and solubility. This was because alkaline conditions shift the solution pH far from myofibrillar proteins’ isoelectric point (pI ≈ 5), increasing myofibrillar proteins’ surface negative charge density. Reduced electrostatic repulsion between myofibrillar protein molecules promoted their proximity and cross-linking, ultimately forming a denser, more ordered 3D gel network (consistent with the microstructural results in Figure 5). This network explained the superior gel strength and WHC of L-Arg and L-Lys groups (Figure 1B,D). According to references, the gel strength of Pacific whiting surimi gels continued to increase with the pH increasing from 6 to 10. This suggested that highly alkaline conditions might induce protein structural unfolding and expose more charged regions that could bind water molecules, thereby improving gel strength and WHC [70]. Moreover, Shi et al. [71] further demonstrated that the improvement in bighead carp myosin gelation arises from the synergistic effect of pH and L-Arg’s molecular structure, where the guanidinium group was critical for conformation modulation and aggregation inhibition. This indicated that the strong alkaline environment caused by the addition of alkaline amino acids is an important factor in enhancing the gel properties, but it is not the sole determining factor. The structure of the amino acids themselves is also of great significance. For L-Pro, the initial pH value of the environment (6.38) was higher than its isoelectric point (pI = 6.30), which might indicate that L-Pro carried a net negative charge and acted as a proton donor [72], thereby slightly acidifying the solution and lowering the pH value. Consequently, the high alkaline properties and specific molecular structure of L-Arg and L-Lys concurrently promoted protein molecular cross-linking, generating a tighter and more regular spatial gel network.

### 3.11. Solubility and Turbidity of Myofibrillar Protein Solution with Various Amino Acids

Solubility indicates the dissolution content of myofibrillar proteins, which serves as the fundamental framework for constructing the three-dimensional gel network [40]. Figure 7B presents the solubility characteristics of myofibrillar proteins with various amino acids. Relative to the LC group (23.64%), L-Lys, L-Arg, and L-Pro groups led to a substantial rise in solubility, reaching 89.58%, 78.20%, and 28.64%, respectively. Notably, L-Lys and L-Arg raised the pH of the myofibrillar protein solution above its isoelectric point, thereby enhancing the binding capacity of proteins and water molecules and markedly improving protein solubility [19]. L-Pro likely shielded hydrophobic protein regions and enhanced interactions between hydrophilic moieties and surrounding residues, suppressing insoluble aggregate formation and promoting protein solubility [16]. However, an excessively high degree of solubility might indicate that protein molecules are overly dispersed, leading to insufficient intermolecular contact and cross-linking, and consequently a sparse network of junction points.

Turbidity can be used to monitor the extent of protein aggregation, reflecting the size and quantity of insoluble suspended particles within the solution [62]. Figure 7C illustrates the turbidity of myofibrillar proteins with various amino acids. L-Arg, L-Lys, and L-Pro groups were all effective in reducing turbidity. The pronounced effect of alkaline amino acids could be attributed to their interaction with proteins, which suppressed aggregation and consequently led to a significant reduction in turbidity [73]. L-Pro enhanced the solubility of myofibrillar proteins [16], which in turn suppressed insoluble aggregate formation and decreased turbidity. Therefore, the concomitant increase in solubility and decrease in turbidity jointly demonstrated that L-Arg, L-Lys, and L-Pro promoted protein interactions, consistent with the findings on gel strength (Figure 1B) and WHC (Figure 1D).

### 3.12. Molecular Docking of L-Arg, L-Lys, and L-Pro with Pacific White Shrimp Myosin

**Figure 8:**
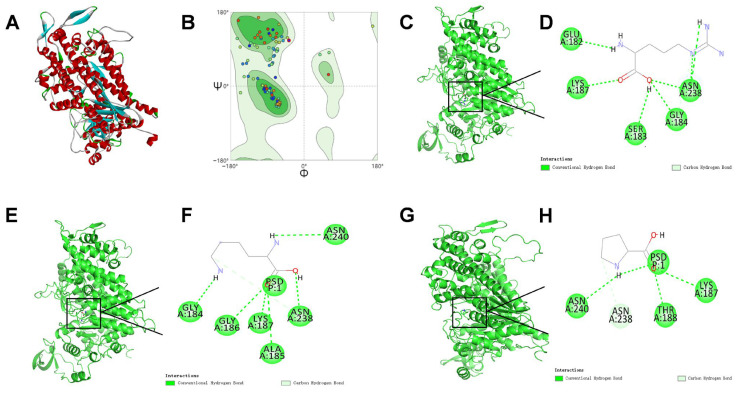
Molecular docking results: (**A**) 3D structure of Pacific white shrimp myosin modeled with homology; (**B**) the Ramachandran plot validated the rationality of the predicted structure of Pacific white shrimp myosin; (**C**,**D**) the binding of L-Arg to Pacific white shrimp myosin was simulated by molecular docking; (**E**,**F**) the binding of L-Lys to Pacific white shrimp myosin was simulated by molecular docking; (**G**,**H**) the binding of L-Pro to Pacific white shrimp myosin was simulated by molecular docking.

Surimi is a multi-component protein system (myosin, actin, tropomyosin, troponin, sarcoplasmic proteins, etc.) and the present docking study was intentionally restricted to myosin because of its quantitative and functional dominance in heat-induced gelation [28]. To examine potential interaction modalities of L-Arg, L-Lys, and L-Pro with myofibrillar proteins, this study utilized the myosin from Pacific white shrimp as the receptor and performed homology modeling and molecular docking analysis. Firstly, the three-dimensional structure of myosin was constructed through homology modeling as a predictive model (Figure 8A). The template protein exhibited a sequence similarity of 61% to the target, with a GMQE score of 0.65, suggesting that the selected template was appropriate and the model quality was acceptable [74]. Further evaluation of the model via the Ramachandran plot (Figure 8B) predicted that 95.61% of the residues resided in the core and additionally allowed regions, supporting the homology model of favorable structural quality that was suitable for further docking analysis. Figure 8C–H show the predicted binding sites of L-Arg, L-Lys, and L-Pro on myosin, along with 2D schematic diagrams. Computational docking analyses predicted minimum binding energies of −6.5, −6.0, and −5.9 kcal/mol for the respective amino acid complexes. These negative values suggest that the binding processes for all three amino acids could be spontaneous, with the predicted binding affinity following the order L-Arg > L-Lys > L-Pro. Visualization of the docking results using PyMOL 2.6.2, combined with analysis of the two-dimensional interaction diagrams (Figure 8D,F,H), suggested that hydrogen bonding might serve as a key stabilizing force in the predicted complexes. Specifically, L-Arg was predicted to form hydrogen bonds with GLU-182, LYS-187, SER-183, GLY-184, and ASN-238 of myosin. L-Lys was predicted to form hydrogen bonds with GLY-184, GLY-186, LYS-187, ALA-185, ASN-238, and ASN-240 of myosin. L-Pro was predicted to form hydrogen bonds with ASN-240, ASN-238, THR-188, and LYS-187 of myosin. In conclusion, the molecular docking analysis hypothesized that all three amino acids could associate with myosin by forming predicted hydrogen-bonding networks, with ASN-238 and LYS-187 identified as potential common interaction sites. In the future, we will conduct further investigations into the interactions between amino acids and other (“non-myosin”) proteins.

### 3.13. Principal Component Analysis

**Figure 9:**
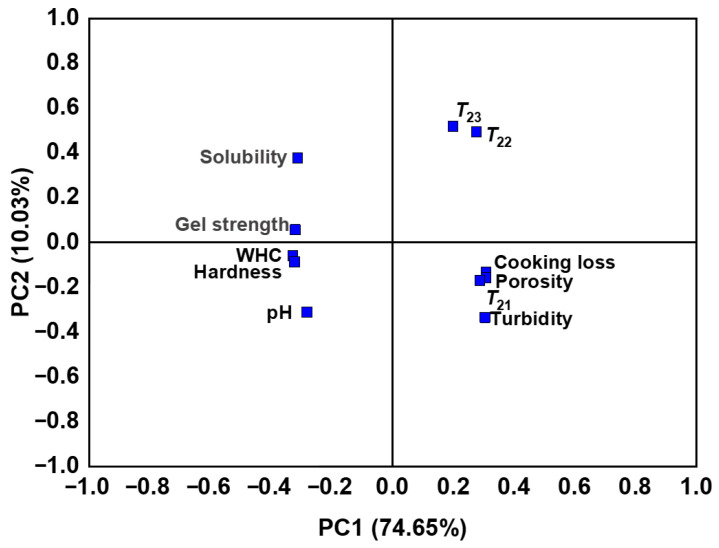
Principal component analysis (PCA) of low-salt SSG with different amino acids.

Principal component analysis (PCA) visualizes the correlations among samples and variables [75]. The PCA biplot in Figure 9 summarizes the gel properties of low-salt SSG. Principal Component 1 (PC1) and Principal Component 2 (PC2) accounted for 74.65% and 10.03% of the variance, respectively, with a cumulative contribution of 84.68%, suggesting that the two-dimensional PCA plot sufficiently represented the majority of the systematic variation in the dataset. PC1 comprised gel strength, hardness, WHC, cooking loss, *T*_21_, *T*_22_, *T*_23_, porosity, pH, solubility, and turbidity. The gel characteristics of low-salt SSG were distributed across all four quadrants. The graph revealed a positive correlation between gel strength and hardness, WHC, solubility, and pH. Conversely, it showed negative associations with another set comprising porosity, cooking loss, turbidity, and the transverse relaxation times *T*_21_, *T*_22_, and *T*_23_, which were all located on the opposite side of PC1.

## 4. Conclusions

The gel characteristics and in vitro digestion properties of low-salt SSG were largely dependent on the intrinsic properties of exogenous amino acids. L-Arg, L-Lys, and L-Pro significantly enhanced gel strength, improved water-holding capacity and moisture distribution, and resulted in a more uniform three-dimensional microstructure, which was attributed to increased protein solubility and the formation of a denser gel matrix. Moreover, the reduced digestibility of L-Lys group suggested that its more stable network might hinder the diffusion of pepsin and trypsin, thereby slowing the digestion process. These three amino acid residues were likely to establish stable interactions with myosin through an extensive hydrogen-bonding network, with ASN-238 and LYS-187 potentially serving as common anchoring sites. These findings provide profound theoretical insights into the precise regulation of surimi gel quality based on amino acid properties, supporting the development of novel low-salt shrimp surimi products. Future work should investigate both the nutritional profile (e.g., amino acid composition) of digested low-salt shrimp surimi products and the effects of equimolar concentrations of key components, to clarify the relationship between molecular stoichiometry, functional effects, and ultimate nutritional quality in surimi systems.

## Figures and Tables

**Table 1:** Effects of different amino acids on TPA (hardness, springiness, cohesiveness, chewiness) of low-salt SSG.

Type of Amino Acid	Hardness (g)	Springiness	Cohesiveness	Chewiness (g)
LC	1613.57 ± 60.40 ^d^	0.81 ± 0.06 ^c^	0.64 ± 0.07 ^b^	691.84 ± 56.06 ^e^
L-Arg	2172.92 ± 48.15 ^b^	0.95 ± 0.04 ^a^	0.77 ± 0.01 ^a^	1457.46 ± 65.58 ^b^
L-Lys	2374.18 ± 99.60 ^a^	0.94 ± 0.03 ^a^	0.77 ± 0.01 ^a^	1686.02 ± 45.31 ^a^
L-Pro	1910.03 ± 55.52 ^c^	0.87 ± 0.04 ^bc^	0.65 ± 0.02 ^b^	814.69 ± 56.63 ^d^
HC	1902.27 ± 89.70 ^c^	0.93 ± 0.02 ^ab^	0.71 ± 0.01 ^a^	1170.37 ± 57.61 ^c^

Different lowercase letters in the same column denote significant differences (*p* < 0.05).

**Table 2:** Effects of different amino acids on the water status of low-salt SSG.

Types of Amino Acids	*T*_21_ (ms)	*T*_22_ (ms)	*T*_23_ (ms)
LC	2.89 ± 1.37 ^a^	55.86 ± 2.38 ^ab^	1113.94 ± 125.99 ^a^
L-Arg	0.85 ± 0.06 ^b^	42.76 ± 1.78 ^c^	961.00 ± 78.85 ^bc^
L-Lys	0.58 ± 0.22 ^b^	41.73 ± 1.78 ^c^	871.50 ± 63.50 ^c^
L-Pro	2.17 ± 0.80 ^ab^	54.49 ± 0.00 ^b^	893.44 ± 73.30 ^c^
HC	1.22 ± 1.23 ^ab^	58.61 ± 0.00 ^a^	1082.66 ± 0.00 ^ab^

Different lowercase letters in the same column denote significant differences (*p* < 0.05).

**Table 3:** Effects of different amino acids on secondary structure of low-salt SSG.

Type of Amino Acid	α-Helix	β-Sheet	β-Turn
LC	36.87 ± 1.08 ^d^	39.74 ± 3.15 ^c^	23.39 ± 2.08 ^a^
L-Arg	47.20 ± 0.09 ^b^	50.47 ± 0.13 ^ab^	2.33 ± 0.07 ^b^
L-Lys	48.87 ± 0.07 ^a^	48.25 ± 0.12 ^b^	2.88 ± 0.07 ^b^
L-Pro	37.79 ± 0.16 ^d^	41.66 ± 4.26 ^c^	20.55 ± 4.36 ^a^
HC	44.00 ± 0.12 ^c^	53.70 ± 0.23 ^a^	2.29 ± 0.10 ^b^

Different lowercase letters in the same column denote significant differences (*p* < 0.05).

**Table 4:** Effects of different amino acids on the relative intensity of Raman spectra of low-salt SSG.

Type of Amino Acid	LC	L-Arg	L-Lys	L-Pro	HC
I_850_/I_830_ I_760_/I_1003_	0.99 ± 0.03 ^ab^ 0.45 ± 0.05 ^a^	0.97 ± 0.01 ^bc^ 0.27 ± 0.01 ^c^	0.94 ± 0.01 ^c^ 0.36 ± 0.04 ^b^	1.01 ± 0.01 ^a^ 0.42 ±0.01 ^ab^	0.95 ± 0.02 ^c^ 0.46 ± 0.03 ^a^

Different lowercase letters in the same column denote significant differences (*p* < 0.05).

## Data Availability

The data presented in this study are available on request from the corresponding author.

## Supplementary Materials

The following supporting information can be downloaded at https://www.mdpi.com/article/10.3390/foods15020400/s1, Figure S1: Effects of different amino acids at different concentrations (0.5% and 1%) on gel strength of low-salt SSG.; Figure S2: Effects of different amino acids on breaking deformation (A) and breaking force (B) of low-salt SSG.

## Abbreviations

The following abbreviations are used in this manuscript:

L-Arg	L-arginine
L-Lys	L-lysine
L-Pro	L-proline
LC	low-salt control
HC	high-salt control
SH	sulfhydryl
