# Supplementary material for: Roles of Amino Acid Properties in Regulating the Gel Characteristics of Low-Salt Pacific White Shrimp (Litopenaeus vannamei) Surimi
Source: Foods. 2026 Jan 22;15(2):400. doi: 10.3390/foods15020400 (PMC12840827; doi:10.3390/foods15020400)
Supplement: Supplementary file 1 [file foods-15-00400-s001.zip › foods-4053952-supplementary.pdf]

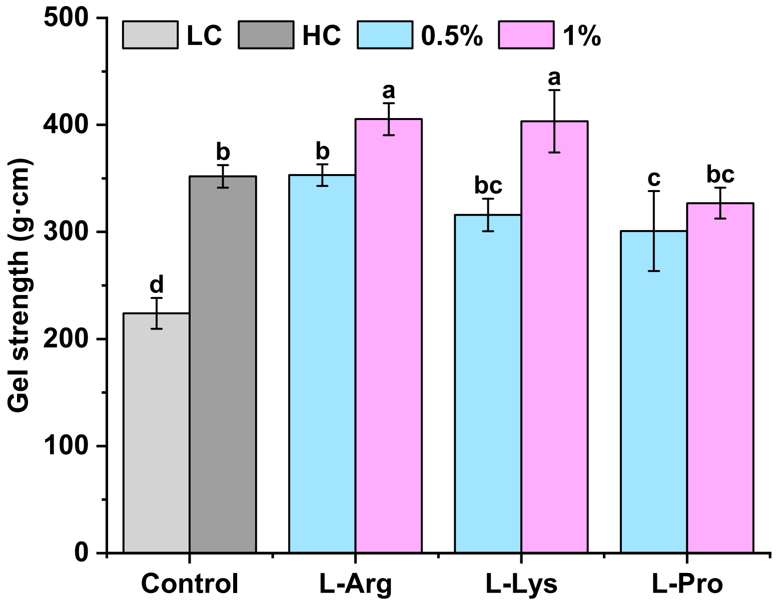

Figure S1. Effects of different amino acids at different concentrations (0.5% and 1%) on gel strength of low-salt SSG. Different lowercase letters in the same index denote significant differences ($P < 0.05$).

A B

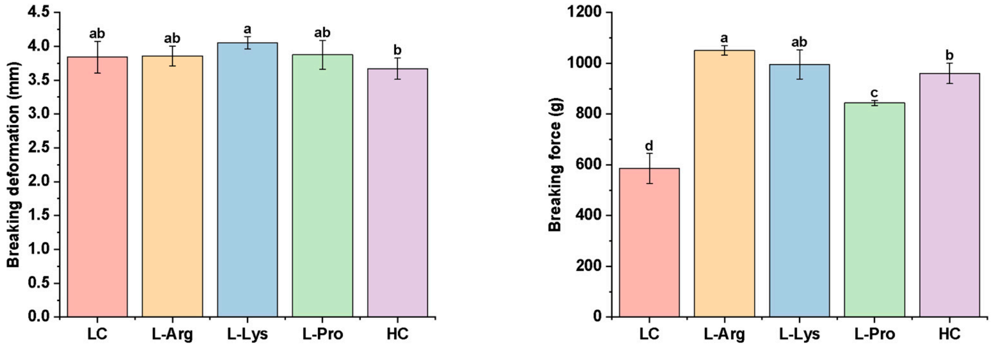

Figure S2. Effects of different amino acids on breaking deformation (A) and breaking force (B) of low-salt SSG. Different lowercase letters in the same index denote significant differences ($P < 0.05$).
